# Epigenetic embedding of childhood adversity: mitochondrial metabolism and neurobiology of stress-related CNS diseases

**DOI:** 10.3389/fnmol.2023.1183184

**Published:** 2023-07-25

**Authors:** Benedetta Bigio, Yotam Sagi, Olivia Barnhill, Josh Dobbin, Omar El Shahawy, Paolo de Angelis, Carla Nasca

**Affiliations:** ^1^Department of Psychiatry, New York University Grossman School of Medicine, New York, NY, United States; ^2^Center for Dementia Research, Nathan S. Kline Institute for Psychiatric Research, Orangeburg, NY, United States; ^3^Harold and Margaret Milliken Hatch Laboratory of Neuroendocrinology, Rockefeller University, New York, NY, United States; ^4^Department of Population Health, New York University Grossman School of Medicine, New York, NY, United States; ^5^Department of Neuroscience and Physiology, New York University Grossman School of Medicine, New York, NY, United States

**Keywords:** hippocampus, acetyl-L-carnitine, glutamate, histone, mood disorders, depression, cognition, opioid use disorders

## Abstract

This invited article ad memoriam of Bruce McEwen discusses emerging epigenetic mechanisms underlying the *long and winding road* from adverse childhood experiences to adult physiology and brain functions. The conceptual framework that we pursue suggest multidimensional biological pathways for the rapid regulation of neuroplasticity that utilize rapid non-genomic mechanisms of epigenetic programming of gene expression and modulation of metabolic function via mitochondrial metabolism. The current article also highlights how applying computational tools can foster the translation of basic neuroscience discoveries for the development of novel treatment models for mental illnesses, such as depression to slow the clinical manifestation of Alzheimer’s disease. Citing an expression that many of us heard from Bruce, while “It is not possible to roll back the clock,” deeper understanding of the biological pathways and mechanisms through which stress produces a lifelong vulnerability to altered mitochondrial metabolism can provide a path for compensatory neuroplasticity. The newest findings emerging from this mechanistic framework are among the latest topics we had the good fortune to discuss with Bruce the day before his sudden illness when walking to a restaurant in a surprisingly warm evening that preluded the snowstorm on December 18th, 2019. With this article, we wish to celebrate Bruce’s untouched love for Neuroscience.

## Mitochondrial metabolism and epigenetic function

Epigenetic mechanisms are involved in the pathophysiology of stress-related diseases, including depressive and cognitive disorders as well as opioid and alcohol use disorders, and are emerging as potential targets for therapeutic interventions(Sweatt, 2010; Robison and Nestler, 2011; Nestler, 2014; McEwen et al., 2015). Work from our group introduced the McEwen lab to acetyl-L-carnitine (LAC), a central mitochondrial metabolite that was best known for its role in fatty acid oxidation (Fritz and McEwen, 1959; Pettegrew et al., 2000; McEwen et al., 2015; McEwen, 2018). In rodents, administration of LAC leads to a rapid and persistent antidepressant-like response by activating histone acetyltransferases (e.g.: P300) to regulate histone acetylation and expression of key genes, including the metabotropic glutamate receptor-2 (mGlu2, inhibitor of spontaneous glutamate release) and the downstream brain-derived neurotrophic factor BDNF (Flight, 2013; Nasca et al., 2013; Russo and Charney, 2013; Nasca et al., 2017). Boosting mitochondrial metabolism of LAC also leads to the amelioration of specific cognitive domains (Barnes et al., 1990; Liu et al., 2002). These potent epigenetic effects of LAC occur in brain areas such as the hippocampus, which is implicated in the pathophysiology of major depressive disorders (MDD) and is among the first brain structures to degenerate in Alzheimer’s disease (AD) (Braak et al., 1993; McEwen et al., 2016). Furthermore, the rapid effects of LAC extend to other key brain areas important not only for mood disorders but also for substance abuse disorders such as the prefrontal cortex, nucleus accumbens and amygdala (Nasca et al., 2013; Lau et al., 2017; Cherix et al., 2020). In rodent models (e.g., mice after exposure to chronic stress or the flinders sensitive genetic line FSL rats), peripheral and central (e.g., in the hippocampus and prefrontal cortex) LAC levels are decreased; and boosting mitochondrial metabolism of LAC rapidly regulates a metabolic dysfunction known as insulin resistance (IR) (Eriksson et al., 2012; Nasca et al., 2013; Bigio et al., 2016). In recent years, this mechanistic framework in rodents led to test novel hypotheses in humans in pursuit of developing disease-modifying drugs and precision medicine strategies for stress-related main CNS diseases.

## From basic neuroscience discoveries to translational research

In subjects suffering from MDD, LAC levels are decreased as compared to age-and sex-matched controls; the degree of LAC deficiency reflected both the severity and age of onset of depression (Nasca et al., 2018; Post, 2018; Nasca et al., 2020). We found the lowest levels of LAC in severe clinical phenotypes of treatment resistant depression associated with early life stress in the form of childhood emotional trauma. Utilization of esketamine as an antidepressant increases LAC levels (Rotroff et al., 2016). Decreased levels of LAC are also predictive of lack of antidepressant responses to the insulin-sensitizing agent pioglitazone used as an antidepressant in subjects suffering from MDD (Nasca et al., 2021). Recently, we also showed a relationship between the epigenetic modulation of glutamatergic function and central IR as assessed by measures of the insulin signaling cascade in exosomes enriched for the neural cell adhesion molecule L1 (L1-CAM), a protein highly expressed in the brain (Takahashi et al., 2012; Lonsdale et al., 2013; Uhlen et al., 2015; Nasca et al., 2020). As we and others reviewed elsewhere (Kenna et al., 2013; Biessels and Reagan, 2015; Grillo et al., 2015; Rasgon and McEwen, 2016; Arnold et al., 2018; Ferrario and Reagan, 2018; Watson et al., 2018), in addition to systemic energy metabolism, insulin signaling contributes to regulate neuroplasticity and is reflective of cerebral hypo-metabolism and aberrant intrinsic connectivity of intra-and inter-hippocampal circuits. A growing literature suggests that IR—which is ameliorated by boosting mitochondrial metabolism of LAC in rodent models—is one of the steps in the irreversible activation of the cascade leading from mood disorders to AD (Byers and Yaffe, 2011; De Felice et al., 2014; Rasgon and McEwen, 2016; De Felice et al., 2022).

In the connection between mitochondrial metabolism and aging, prior work reported decreased levels of LAC in subjects with AD as compared to cognitively healthy controls, with intermediate levels in subjects with subjective memory complaint or mild cognitive impairment (MCI) (Cristofano et al., 2016). As we elaborated above, these translational findings are an outgrowth of a mechanistic model in rodents with impaired plasticity of key brain areas relevant to mood, cognitive, opioid and alcohol use disorders, wherein LAC levels are markedly decreased and signal abnormal brain and systemic functions. The current mechanistic model compels further research to identify new signaling pathways and mechanisms for developing novel treatment models for mental illness, ultimately to slow the clinical manifestation to dementia (Byers and Yaffe, 2011; Rasgon and McEwen, 2016).

## Early life stress and adverse childhood experiences

At the same time, while we continue to learn the role of multidimensional biological pathways—which utilize rapid non-genomic mechanisms of epigenetic regulation of gene expression and modulation of metabolic function—in neuroplasticity (McEwen et al., 2015), there is increasing recognition that adverse childhood experiences disproportionately influence lifelong vulnerability to develop mood and cognitive disorders (McEwen, 2003; McEwen et al., 2015; Nemeroff, 2016). Prior gene expression studies showed a brain that continually changes with experience and that the biological embedding of trajectories of neuroplasticity starts in early life (Shonkoff et al., 2009; Gray et al., 2014). Adverse experiences, such as decreased maternal care early in life, leads to lifelong changes in the epigenome (e.g.: histone acetylation) and the related expression of key genes for the responses to stress in the hippocampus; and these changes are accompanied by behavioral deficits (Meaney et al., 1988; Weaver et al., 2004). Regarding excessive glutamate overflow, mice with inherent anxiety at baseline show elevated expression of the mineralocorticoid receptors (MR) in the hippocampus that predisposes to a stress-induced suppression of mGlu2 expression and development of depressive-like behavior. Blocking MR receptors and interfering with glucocorticoids stimulation of glutamate activity counteracts stress-induced behavioral abnormalities. Yet, the nature of the experiences of the animals that develop increased MR expression is not known but might involve epigenetic experiences early in life, such as maternal care and stressors in the neonatal nesting environment as we fully described in the epigenetic allostasis model (Nasca et al., 2015).

Exposure to early life stress also leads to a decrease in hippocampal volume in adult subjects suffering from MDD (Saleh et al., 2017). Recent studies also showed a decreased hippocampal volume in children with depression (Barch et al., 2019; Zovetti et al., 2022). Timing of the stress is found to negatively affect hippocampal volume; the strongest effects were found when stress exposure occurred before 5 years of age (Gerritsen et al., 2015; Humphreys et al., 2019). Anxiety and co-dependence of the mother during the first weeks after birth also resulted in long-lasting effects on the hippocampal volume in young adult offspring, as well as in children from low socioeconomic status households (Hanson et al., 2015; Mareckova et al., 2018). In the connection to metabolism, studies in humans showed that childhood trauma is not only a risk factor for aberrant mitochondrial metabolism in severe clinical phenotypes of treatment resistant depression, but also for IR as well as for shortening of leukocyte telomere length (LTL, a marker of cellular aging) (Price et al., 2013; Nasca et al., 2018; Post, 2018; Nasca et al., 2021). We reported that emotional trauma is a critical factor for the decreased LAC levels in severe clinical phenotypes of treatment resistant depression (Nasca et al., 2018; Post, 2018). We showed a relationship between emotional trauma, but none of the other subscales of the childhood trauma, and antidepressant responses as a function of LAC levels and the corresponding IR and LTL (Nasca et al., 2021). The specificity of these effects is in agreement with prior studies showing that the consequences of emotional maltreatments in childhood differ from those of physical and sexual abuse (Nemeroff, 2016; Williams et al., 2016). There is also considerable evidence that childhood trauma, particularly emotional maltreatment, impairs responses to antidepressant drugs (Nemeroff et al., 2003; Nemeroff, 2016). Although there are fewer studies describing how specific subscales of childhood trauma affect the responses to drugs that ameliorate cognitive function, prior work suggested that adverse childhood experiences result in poorer response to cognitive-behavioral therapy (Short and Baram, 2019). Collectively, the current work raises the hypothesis for future studies that targeting mitochondrial metabolism opens windows of epigenetic plasticity to re-direct the life course trajectories toward more positive health outcomes when adverse childhood experiences occurred.

## Importance of the anterior (human) or ventral (rodent) hippocampus in stress and CNS disorders

Growing literature showed that the ventral hippocampus (vHIPP) in rodents (anterior hippocampus in humans) is a stress-sensitive circuit and a neural hub key for the regulation of behaviors implicated in depression, such as social interaction and anhedonia, as well as for cognitive functions. The vHIPP is also a target for antidepressant action of rapid acting agents, such as ketamine and LAC (Jett et al., 2015; Carreno et al., 2016). The vHIPP is connected to limbic areas involved in affective, reward and cognitive functions. It receives intense inputs from the ventromedial parts of the entorhinal cortex, carrying information arising from the infralimbic and prelimbic cortices as well as from the ventral tegmental area in the midbrain and the locus coeruleus and raphe nuclei in the brain stem. In turn, the vHIPP sends projections to the prefrontal cortex, nucleus accumbens, amygdala, and hypothalamus (Jonas and Lisman, 2014) among other brain areas. Therefore, plasticity of the ventral hippocampus is key in mediating changes in behaviors and cognitive functions. For example, vHIPP glutamatergic afferents to the nucleus accumbens regulate susceptibility to social defeat stress and the corresponding behavioral responses as shown by optogenetic studies (Bagot et al., 2015).

## Computational approaches and multidimensional predictors of health trajectories

This is a time of enormous technological advance in so many aspects of neuroscience and medicine that is bringing together basic, computational, and clinical laboratories to develop novel mechanism-based treatment models for specific clinical phenotypes of stress-related disorders. We are now increasingly recognizing that heterogeneous psychiatric disorders, such as MDD, are likely much more biologically distinct than are captured by self-report symptom clusters. Using functional magnetic resonance imaging (fMRI), recent work showed that subjects suffering from depression can be subdivided into four neurophysiological subtypes defined by distinct patterns of dysfunctional connectivity in limbic and frontostriatal networks (Drysdale et al., 2017). Subtypes 1 and 4 are mainly characterized by reduced connectivity of frontoamygdala and increased anxiety; subtypes 3 and 4 are characterized by hyper-connectivity of thalamic and frontostriatal networks with increased anhedonia and psychomotor retardation. The four biotypes also predicted different antidepressant responses to repetitive transcranial magnetic stimulation. Toward understanding of the molecular mechanisms that might characterize the four neurophysiological subtypes, recent work showed that boosting mitochondrial metabolism leads to a rapid amelioration of anhedonia-related behaviors in rodent models deficient in LAC, which we now know is also a factor in clinical phenotypes of treatment resistant depression, suggesting it might be a potential treatment target, especially for these specific biotypes (Nasca et al., 2013, 2018). It is also important to note that subjects with opioid use disorders, particularly those in methadone and buprenorphine treatment programs, manifest a significant anhedonia which is in turn linked to poorer responses to treatment for tobacco use (Leventhal et al., 2009; Roys et al., 2016; Cook et al., 2017; Parker et al., 2020; Streck et al., 2020). These findings compel further research to understand whether certain aspects of mitochondrial metabolism and the related anhedonic states might serve as new targets for the development of more effective mechanism-based treatment models for psychiatric and substance abuse disorders.

Using hierarchical clustering to integrate molecular measures with clinical symptoms in those patients suffering from MDD characterized by a LAC deficiency, we also found that specific symptoms related to anhedonia, depressed mood, feelings of guilt, and suicidality are accompanied by a brain metabolic dysfunction known as IR as showed by an increase and sex-specific phosphorylation in the expression of IRS1, a key marker of the insulin signaling cascade, in discrete exosomes enriched for the brain (Nasca et al., 2020). These findings provided the closest available *in vivo* molecular signature for brain IR in depression and showed important sex differences in these pathways. In keeping with the important role of childhood trauma on adult physiology and brain functions, prediction profile modelling revealed that those patients suffering from MDD and characterized by decreased baseline LAC levels, elevated BMI and high reported rates of emotional abuse show the worst antidepressant responses; conversely, those patients with increased baseline LAC levels, decreased BMI and low reported rates of emotional abuse show decreased depression severity at the HDRS-21 in the responses to the insulin-sensitizing agent pioglitazone used as an antidepessant (Nasca et al., 2021). These recent findings suggest that multidimensional factors spanning mitochondrial metabolism, cellular aging, metabolic function, and childhood trauma provide more detailed signatures to predict antidepressant responses. Integrating molecular and circuit-level approaches has the potential to transform our understanding of the molecular underpinning of circuit-level abnormalities, and inform future efforts to develop personalized interventions with the potential for significantly enhanced efficacy compared to the current standard-of-care.

An additional application of computational tools includes the integration of multidimensional phenotypic measures to identify those mechanisms that predispose apparently healthy individuals to develop maladaptive coping strategies from those that confer resilience. Using a high-throughput unbiased automated phenotyping platform that collects >2000 behavioral features based on machine learning, recent work showed that a rich set of behavioral alterations distinguish susceptible versus resilient phenotypes after exposure to social defeat stress (SDS) (Lorsch et al., 2021). Interrelated brain–body marker characterize these phenotypes before any applied stress. We showed that a subgroup of mice characterized, at baseline, by increased anxiety at the light–dark test, the corresponding elevation of pro-inflammatory cytokine IL-6 as well as smaller hippocampal volume develops behavioral and neurobiological deficits after exposure to SDS, with social withdrawal and impaired transcriptomic-wide changes in ventral dentate gyrus (Nasca et al., 2019). At the individual level, a computational approach used to integrate *in vivo* measures of anxiety and immune system function predicted if a given animal developed SDS-induced social withdrawal, or remained resilient, with a sensitivity of 80% that is stronger than the categorization power based on either individual measure alone (Nasca et al., 2019). The findings of *a priori* multidimensional biomarkers for predicting the behavioral deficits resulting from exposure to SDS suggests a unique approach to examine the individual trajectories of adaptative and maladaptive responses to stress, paving the way to develop integrative models of mechanisms leading to susceptibility versus resilience to stress.

### Future directions: in pursuit of “windows of epigenetic plasticity” to re-direct trajectories of brain function

**Figure 1:**
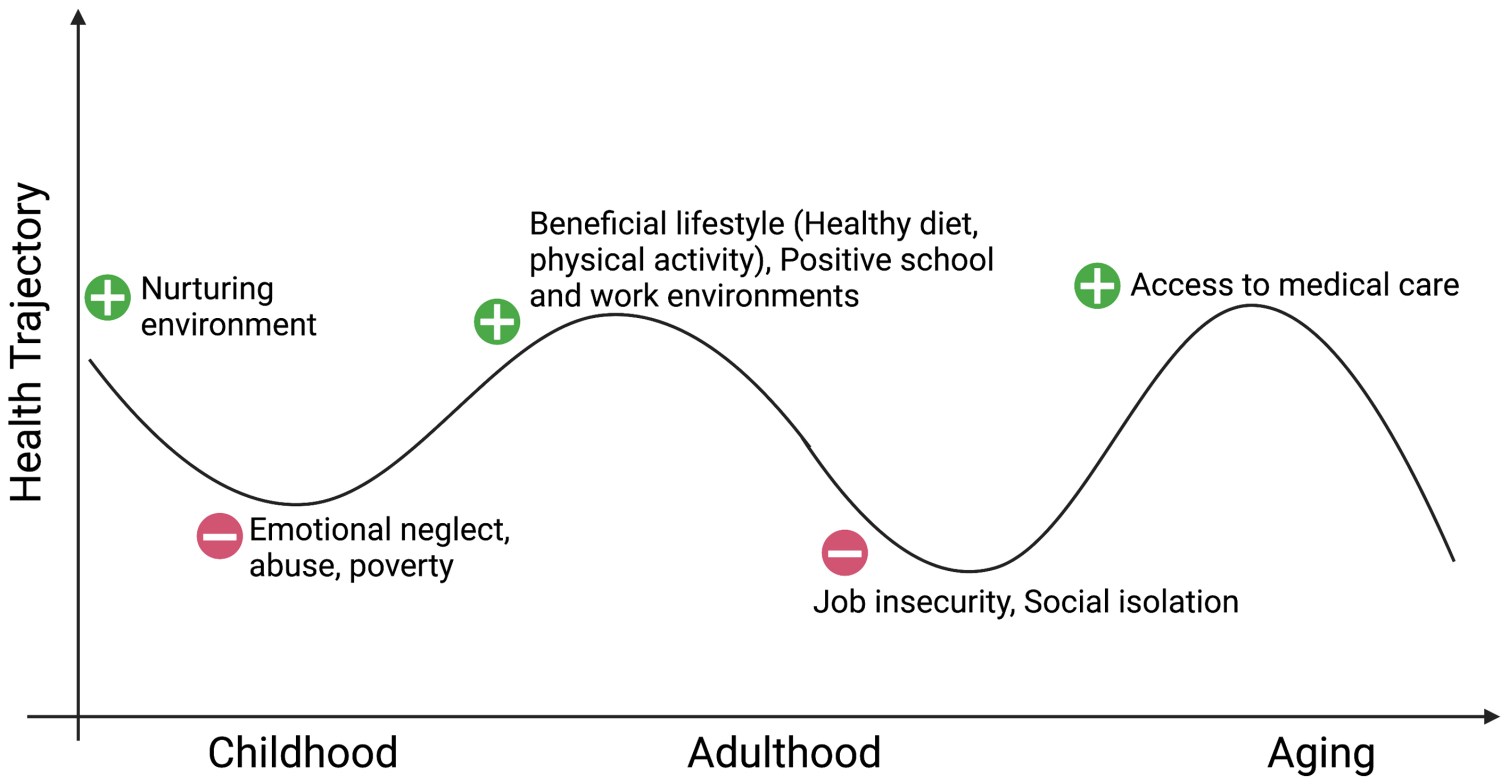
Social environment and health: in pursuit of windows of epigenetic plasticity. Created with BioRender.com.

While prevention is paramount, identifying novel biological pathways and mechanisms through which stress, including childhood trauma, affects adult systemic physiology and brain functions is a timely topic crucial for the development of new mechanistic frameworks to build resilience or decrease vulnerability to main CNS diseases, where adverse events have happened. Beyond recognizing resilience as “achieving a positive outcome in the face of adversity” (McEwen et al., 2015), there appears to be a common denominator in the trajectories to stress-related disorders that we propose involves an epigenetic embedding of early life experiences through the mitochondrial metabolite LAC that, when supplemented, rapidly alters gene expression profiles to ameliorate behaviors and cognitive function in animal models deficient in LAC because of stress-induced causes. The concept of epigenetic embedding of early life experiences is akin to the original definition of epigenetics, wherein the emergence of characteristics of each individual is not evident from prior stages of development (Waddington, 1942). While it is not possible to “roll back the clock,” deeper understanding of the biological pathways and mechanisms through which adverse childhood experiences produce a lifelong vulnerability to altered mitochondrial metabolism can provide a path for compensatory neuroplasticity toward more positive health directions (Figure 1). The COVID-19 pandemic and the related biological and social fallouts further highlight the need for basic neuroscience research to identify new signaling pathways and mechanisms underlying the effects of stress on the brain and the rest of the body to develop mechanistic-based treatment models (COVID-19 Mental Disorders Collaborators, 2021; WHO, 2022).

## Author contributions

CN and BB wrote the manuscript. BB, YS, OB, JD, OES, PA, and CN provided inputs to this article. All authors contributed to the article and approved the submitted version.

## Funding

This work was supported by a R01MH128311, a R56MH125895, and a NARSAD Young Investigator Grant #30051 from the Brain & Behavior Research Foundation to CN, and by the National Institute on Aging (NIA) through the Early Adversity & Later Life Reversibility Pilot Grant to CN under Award Number R24AG06517.

## Conflict of interest

The authors declare that the research was conducted in the absence of any commercial or financial relationships that could be construed as a potential conflict of interest.

## Publisher’s note

All claims expressed in this article are solely those of the authors and do not necessarily represent those of their affiliated organizations, or those of the publisher, the editors and the reviewers. Any product that may be evaluated in this article, or claim that may be made by its manufacturer, is not guaranteed or endorsed by the publisher.
